# A clinical characteristic analysis of pregnancy-associated intracranial haemorrhage in China

**DOI:** 10.1038/srep09509

**Published:** 2015-03-30

**Authors:** Zhu-Wei Liang, Li Lin, Wan-Li Gao, Li-Min Feng

**Affiliations:** 1 Department of Obstetrics and Gynecology, Beijing Friendship Hospital, Capital Medical University, Beijing, 100050, China; 2 Department of Obstetrics and Gynecology, Beijing Tiantan Hospital, Capital Medical University, Beijing, 100050, China

## Abstract

Intracerebral haemorrhage (ICH) occurring during pregnancy and the puerperium is an infrequent but severe complication with a high mortality and poor prognosis. Until recently, previous studies have mainly focused on the effect of different treatments on prognosis. However, few studies have provided solid evidence to clarify the key predisposing factors affecting the prognosis of ICH. In the present study, based on a unique sample with a high ICH incidence and mortality rate, we described the main clinical characteristics of ICH patients and found that the prognosis of patients who underwent surgical intervention was not better than that of patients who received other treatment modalities. However, pre-eclampsia patients had higher maternal and neonatal mortality rates than other aetiology groups. Furthermore, univariate regression analysis identified onset to diagnosis time (O-D time) and pre-eclampsia as the only factors showing independent correlation with poor maternal outcomes (modified Rankin Scale, mRS ≥ 3), and only O-D time was identified as a predictor of maternal mortality. These results revealed that the aetiology of ICH and O-D time might be crucial predisposing factors to prognosis, especially for patients with pre-eclampsia. The study highlighted a novel direction to effectively improve the prognosis of pregnancy-associated ICH.

Pregnancy-associated intracranial haemorrhage (ICH) is an infrequent but severe complication: the estimated mortality of pregnancy-associated ICH is 9–38%^1^,^2^,^3^,^4^,^5^,^6^, which contributes to more than 12% of all maternal deaths in most countries^7^,^8^. The incidence of pregnancy-associated ICH in populations in developing countries is even higher than that reported in other countries; for example, in China, the incidence is as high as 53 cases per 100,000 deliveries^9^. Although the absolute risk of pregnancy-associated ICH is relatively small, it has a significant impact on the prognosis for mother and foetus: its survivors may suffer profound and permanent disability^10^. However, due to the low incidence rate of ICH, neurosurgeons and obstetricians often lack sufficient experience in treating such patients and often fail to make prompt judgments based on changes in patients' condition. Consequently, they cannot remove predisposing factors in a timely manner and effectively improve the prognosis of these patients. In the present study, we attempted to assess the key predisposing factors impacting prognosis of pregnancy-associated ICH.

Until recently, little was known regarding what factors influence the prognosis of ICH in pregnancy or the puerperium. In previous reports, neurosurgeons have mainly focused on whether different treatments for cerebral haemorrhage (i.e., surgery or conservative treatment) have such impact. For example, Dias and Sekhar^10^ reported that early surgical management of aneurysms during pregnancy and the puerperium compared with conservative treatment was associated with lower maternal (63% vs. 11%) and foetal (23% vs. 5%) mortality. Liu et al. reported similar patters but fail to found significant effect of surgical intervention: among 18 cases of ICH during pregnancy and the puerperium, six of the women underwent neurosurgical intervention and the maternal as well as foetal mortality were both 16.67%, whereas 12 received conservative treatment and the maternal and foetal mortality were 46.17% and 45.46%^9^. However, because of the limited, small sample sizes and potential for bias in most previous studies, no solid conclusion can be drawn as to whether surgical intervention is better than conservative treatment. As a result, such prevalence studies are unable to draw a causal distinction between different treatments and other possible key factors that influence the prognosis of ICH patients.

Among these factors, the aetiology of pregnancy-associated ICH (especially pre-eclampsia) is a highly likely but less frequently investigated possibility. Pregnancy-associated ICH is usually caused by pre-eclampsia, eclampsia, or cerebrovascular malformation (CVM), and several studies have even suggested that pregnancy-associated ICH may be a main cause of death in pre-eclampsia patients. Hypertension, which is associated with both ischemic and haemorrhagic stroke, is a primary feature of pre-eclampsia^11^. The general condition of pre-eclampsia patients with cerebral haemorrhage is poor, and both maternal and neonatal mortality rates are higher in such patients than in patients with ICH caused by another aetiology, although these patients only comprise a small proportion of pregnancy-associated ICH patients. A nationwide inpatient sample study reported that pre-eclampsia is associated with a 4-fold increase in stroke during pregnancy in the United States^8^,^12^. The most recent Enquiries into Maternal Death from the United Kingdom showed that the majority of women with pre-eclampsia died from ICH^13^.

In contrast, it is only recently that attention has been paid to the reverse causal relationship between pregnancy-associated ICH and pre-eclampsia, that is, whether pre-eclampsia is a main cause of death in patients with pregnancy-associated ICH. One possible reason of such association is the pathophysiologic features of pre-eclampsia. The basic pathological changes of pre-eclampsia are systemic small artery spasms, vascular endothelial cell damage, and increased brain capillary permeability. Furthermore, plasma and red blood cells can leak into the extravascular space in the brain and cause spotting^14^. The blood pressure is similarly damaging to vessel walls, and it increases sharply when the pressure in the brain's blood vessels increases, which can easily lead to rupture and bleeding. Moreover, pre-eclampsia usually develops quickly; in general, this condition is already serious (e.g., multiple organ failure) upon admission. As a consequence, concurrent cerebral haemorrhage might be the end-stage manifestation of pre-eclampsia, and the presence of clotting during late pregnancy can lead to ICH. These studies together hint that the aetiology of ICH might be one of the factors influencing the outcomes of ICH patients. Recently, Yoshimatsu first noted that pre-eclampsia and hemolysis elevated liver enzymes and low platelet count (HELLP) syndrome may be significantly associated with poor outcomes in pregnancy-associated ICH patients^15^. However, few investigations have directly explored whether and which aetiology influences the prognosis of ICH patients.

In our current research, we aimed at using a unique sample with high ICH incidence and mortality rates to identify the key predisposing factors influencing the prognosis of patients with ICH occurring during pregnancy and the puerperium. We collected data on 39 pregnancy-associated ICH patients. We compared the prognosis of mother and foetus and other clinical characteristics between different treatment and aetiology groups. At last, we identified the predictors of poor maternal outcomes maternal mortality by regression analysis.

## Results

### General clinical features of ICH patients

Baseline demographic variables and the clinical characteristics of the study sample are shown in Table 1. We found that ICH patients have several unique clinical characteristics with respect to factors such as age, aetiology, Glasgow score, gestational age, and obstetric outcome that varied according to disease stage.

#### Age

We found that patients with pregnancy-associated ICH were relatively young. Of the 39 patients, 30 (76.92%) were less than 25 years old, including three patients who were 19 years old. Among this young population of less than 25 years old, the mortality rate of pregnancy-associated ICH was as high as 30.77%, which indicates its dangerous nature.

#### Aetiology

In terms of their prevalence, arteriovenous malformations (AVMs) are likely the most common cause of cerebral haemorrhage (41.03%), followed by pre-eclampsia (including HELLP syndrome, 20.51%); together, these common causes accounted for 61.54% of all cases.

#### Mortality

The mortality rate of the 39 sample patients with pregnancy-associated ICH included in this study was 12 (30.78%), including 5 patients in the surgery group and 7 patients in the conservative treatment group.

#### Glasgow score

The average Glasgow score of patients with pregnancy-associated ICH while admitted to the hospital was 11.77 ± 4.51, and the range was [2, 15]. This result suggests that the neurological symptoms of the vast majority of patients at admission were not severe.

#### Gestational age

The mean gestational age of patients with pregnancy-associated ICH was 29.42 weeks, which varied according to different causes.

#### Delivery method

When sudden ICH occurs during early or mid-pregnancy, patients and their families usually choose abortion, induced labour, or caesarean sections in mid-pregnancy (12 cases, accounting for 30.77% of pregnancy-associated ICH) to ensure the safety of the mothers.

### Comparing the prognosis of different ICH treatment groups

To explore the impact of treatments on the prognosis of ICH patients, we divided the study sample into a surgery group and a conservative treatment group. The aetiology distributions in the two groups were as follows: in the surgery group, 9 AVM, 2 aneurysm, 2 moyamoya disease (MMD), 4 pre-eclampsia, 1 cavernous angioma, 0 cerebral venous sinus thrombosis (CVST), 1 tumour, and 1 unknown reason; in the conservative treatment group, 7 AVM, 2 aneurysm, 2 MMD, 4 pre-eclampsia, 1 cavernous angioma, 2 CVST, 1 tumour, and 0 unknown reasons. Next, we compared the prognosis and other clinical features between the two groups. The results of Wilcoxon-rank testing revealed no significant differences (*P*s > 0.10) between the two groups with respect to age, median gestational age, gravidity, parity, or Glasgow score (Table 2). Similarly, the mortality rates of the mothers, foetuses and neonates between the two groups did not differ (*P*s > 0.10), suggesting that the type of treatment does not affect prognosis.

### Comparing the prognosis of different ICH aetiology groups

To identify the possible effect of aetiology on the prognosis of ICH patients, we divided our sample patients into a pre-eclampsia group and an other aetiology group and compared their prognosis and other clinical characteristics.

First, we identified the clinical characteristics that were significantly different between the two groups. The results of Wilcoxon-rank testing showed that the gestational age of the pre-eclampsia group was significantly higher than that of the other aetiology group (*P* = 0.03). Cerebral haemorrhage caused by pre-eclampsia (including HELLP syndrome) occurred at a later gestational age (35.25 ± 4.10 weeks). The disease period also occurred during the peak period of pre-eclampsia development, which is associated with the progression of the disease characterised by pre-eclampsia. The onset of cerebral haemorrhage caused by other aetiologies occurred at an earlier gestational age (27.75 ± 9.14 weeks), primarily during mid-pregnancy. This result might be related to an intolerance of cerebrovascular diseases caused by the changes in blood volume and cerebral blood flow that occur during the second trimester of pregnancy, including AVM, aneurysm, and cavernous haemangiomas.

Second, we contrasted the prognosis of mother and foetus between the two aetiology groups (Table 3) and found that the prognoses of both mother and foetus in the pre-eclampsia group were lower than those in the other aetiology group. For the prognosis of the mothers, the mortality rate in the pre-eclampsia group was significantly higher than that in the other aetiology group (χ^2^ test, *P* < 0.001). In the pre-eclampsia group, 4 of the 8 patients died due to their cerebral haemorrhage. Two survivors had sudden increases in blood pressure with perinatal proteinuria during childbirth and were diagnosed as having pre-eclampsia complicated by cerebral haemorrhage. The neonates of these patients also died after delivery. For the prognosis of the foetuses and neonates, in the pre-eclampsia group, the mortality rate was significantly higher and the average Apgar score of the newborns was significantly lower than those in the other aetiology group (χ^2^ test, *P* < 0.001).

At last, we performed univariate logistic regression analysis to examine predictors of poor outcome (modified Rankin Scale ≥3) and maternal death. The odds ratios for each risk factor for poor outcome and increased mortality are shown in Table 4. O-D time and pre-eclampsia (including HELLP syndrome) were significantly associated with a poor outcome (*P*s < 0.05). O-D time was also significantly associated with maternal death (*P* < 0.05).

## Discussion

In our current research, we mainly studied the clinical characteristics of pregnancy with ICH. We divided the patients into surgery and conservative treatment groups and found no differences between the maternal and neonatal mortality rates. However, the prognosis of patients with pre-eclampsia was poor compared with ICH patients with other causes. In particular, O-D time and pre-eclampsia (HELLP syndrome) were the only factors that showed independent correlation with poor outcome, and only O-D time showed independent correlation with maternal death. These results suggested that early recognition of ICH can effectively improve the prognosis in patients.

It is worth noting that the studied sample in the current research has some unique features compared with previous studies that endow our work with specific value. First, the incidence rate of ICH during pregnancy and the puerperium in the present study (116.7 cases of pregnancy-associated ICH per 100,000 deliveries) was much higher than that in previous studies. Our result was consistent with previous studies reporting that the condition is more common among Chinese populations^9^. For example, Liu et al.^9^ reported that the incidence of pregnancy-associated ICH in the general Chinese population is 53 cases per 100,000 deliveries. Comparing with these data, the range of previously reported incidence rates (not including China) of pregnancy-associated ICH is 3.8 to 18.1 per 100,000 deliveries^1^,^2^,^3^,^4^,^5^,^6^. Yoshimatsu^7^ recently reported an even lower incidence rate of pregnancy-associated ICH in Japan compared with the above-mentioned data at 3.5 cases per 100,000 deliveries. Second, the mortality rate of ICH during pregnancy and the puerperium in the present study was also higher than in previous studies. The range of the previous reported mortality rate of pregnancy-associated ICH is 9–38%. In Japan, the mortality of such patients is as high as 18.4%. In contrast, in the present study, there were 12 maternal deaths, giving a case mortality rate of 30.77%. These data were consistent with previously reported data in China^9^. One possible reason for these data features is that the Beijing Tiantan hospital in which the studied data were collected is the medical transport centre for pregnant and maternal patients with critical craniocerebral disease in Beijing. As a result, the proportion of ICH patients is relatively higher than in other hospitals.

Based on this unique sample, the present research provided convincing evidence to clarify the impact of surgical treatment on the prognosis of mother and foetus in ICH patients. In comparing the clinical characteristics of the surgery and conservative treatment groups, no significant differences between groups were found for pregnancy-associated ICH. This result substantially differed from the regular clinical practice. For example, Dias and Sekhar^10^ reported that early surgical management of aneurysms during pregnancy and the puerperium was associated with lower maternal (63% vs. 11%) and foetal (23% vs. 5%) mortality compared with conservative treatment. However, Trivedi et al. reported a single case study involving the resection of a ruptured but operable AVM during the pregnancy itself, and this treatment resulted in a positive outcome for both mother and foetus^16^. Till now, a traditional and general treatment plan for ICH patients has been to send patients with more severe cerebral haemorrhage to undergo surgery, while patients with mild symptoms receive conservative treatment. However, as Connolly et al. have addressed, a treatment plan (e.g., deciding whether surgical intervention is warranted) must be determined via a comprehensive assessment of the patient's condition rather than by the severity of the neurological symptoms alone^17^. Patients with mild symptoms might achieve positive results using conservative neurological treatments; however, some patients might miss the best time window for surgical treatment because of their critical condition and may then only be eligible to receive conservative treatment.

Furthermore, the present study demonstrated that a different cause or aetiology may also be a less thoroughly studied factor in previous reports on the prognosis of mother and foetus in ICH patients. The comparison between the pre-eclampsia and other aetiology groups showed that both mothers and their neonates had a high mortality rate if pre-eclampsia (including HELLP syndrome) caused the critical ICH. This result is consistent with the conclusions of Yoshimatsu et al^15^. Concurrent cerebral haemorrhage might be the end-stage manifestation of pre-eclampsia; the presence of clotting during late pregnancy can lead to brain haemorrhage. The present study demonstrated that pre-eclampsia was significantly associated with a poor outcome in mothers. More importantly, O-D time may be one of the most important factors affecting the prognosis, not only with respect to overall poor outcomes but also for maternal mortality. These results highlight a new possible clinical treatment for ICH patients: if the clinician can make all efforts to shorten O-D time, they may effectively improve the prognosis of ICH patients.

Based on this point, we can make a thorough interpretation of the reasons why ICH patients generally have a poor prognosis despite undergoing active treatment. In different stages of pregnancy, the possible reasons for a delayed diagnosis could be as follows. First, *in the prenatal examination stage*. These patients usually ignore prenatal care or delay their disease diagnosis. These patients should receive a detailed pre-pregnancy check and a detailed assessment regarding their ability to become pregnant and should be closely monitored during pregnancy; furthermore, the prenatal care intervals should be shortened, changes in their condition should be detected at an early stage, and pregnancies should be terminated when necessary. Second, *in the* *in-hospital treatment stage*. ICH patients with onset during pregnancy are more likely to be neglected by their obstetricians for the following potential reasons: 1) The early symptoms of ICH in patients (e.g., vomiting) are likely be mistaken for morning sickness and erroneously ignored by the obstetrician. 2) The early symptoms of ICH in patients are likely to be confounded by symptoms of pre-eclampsia caused by elevated blood pressure, which may also include symptoms such as dizziness, headache, and vertigo. 3) Some ICH patients may initially experience atypical symptoms such as a high fever that could be misdiagnosed as a puerperal infection and be given anti-inflammatory treatment by their obstetrician. With these atypical conditions, the true illness is generally found later than in cases with typical presentations. Of the patients in the current study, one patient belonged to this group and later died. 4) The ICH symptoms may be masked by the side effects of ICH in the treatment of pre-eclampsia such as dizziness, vertigo, and vomiting. These side effects may be caused by treating pre-eclampsia with an infusion of magnesium sulphate. 5) After delivery, the condition of pre-eclampsia patients with high blood pressure might be delayed by lingering sedative effects. To ensure that such patients achieve sufficient rest, some obstetricians used to give them sedative infusions at 4 to 6 hour intervals. As a result, if those patients fall unconscious, their condition may not be noticed until the drug is stopped. 6) The incidence of ICH is relatively low, so obstetricians in an ordinary hospital may only encounter one or two cases in their career.

The present results have several clinical implications. First, multidisciplinary care is needed in the treatment of such patients. This finding shows that it is crucial to individualise treatment plans based on patient condition. Thus, therapists, including obstetricians, neurologists, neurosurgeons, and ICU physicians should collaborate to provide high-quality, timely, and effective treatment. Second, obstetricians should further their knowledge of ICH. Generally, obstetricians treat patients with ICH that is mainly caused by pre-eclampsia and whose prognosis is relative poor relative to other ICH patients. Thus, updating obstetricians' knowledge of ICH will improve the rate of early detection of ICH. Third, the current study highlighted the urgent need to develop medical transport centres for ICH patients, especially in developing countries. The medical transport centre should be qualified to have extensive and specialised experience with certain diseases. For most general hospitals, severe cases of ICH in maternal patients are relatively rare, and they seldom have sufficient experience in treating such patients. With such well-established medical transport centres, clinicians can promptly transport such patients, which can effectively improve the success rate and reduce the maternal and neonatal mortality rates.

This study suffers from several limitations. First, although the sample size is much larger than in previous reports, it is still not sufficiently large to generalise broadly based on our results. Second, the reported rate of morbidity and mortality of ICH patients in the studied sample might have tended to be higher than in a general population because the studied sample was restricted to a single hospital, Tiantan Hospital, which is the medical transport centre for pregnant and maternal patients with critical craniocerebral disease in Beijing. However, it is still possible that the screening of sample patients missed a few cases. Third, the current study did not study the prognosis of newborns over a longer timeframe.

In summary, we revealed that the prognosis of patients undergoing surgical intervention was not better than that of patients receiving other treatment methods. However, we found that pre-eclampsia patients had higher maternal and neonatal mortality rates compared with the other aetiology group. The present study demonstrated that pre-eclampsia was significantly associated with a poor outcome in mothers. Furthermore, O-D time positively correlated with a poor outcome and maternal death. This study highlighted a novel direction for clinicians to effectively improve the prognosis of pregnancy-associated ICH, that is, to shorten the O-D time of ICH patients and identify ICH patients with pre-eclampsia in a timely manner.

## Methods

The study was approved by the institutional review committee. Participants were retrospectively screened from a database containing the records of 33,416 pregnant women who delivered at Beijing Tiantan Hospital, China, between January 1997 and December 2012. The inclusion criteria set as follows: All the participants were pregnant or within 6 weeks postpartum, and all cases of pregnancy-associated ICH were verified by cranial computed tomography scan. Patients with suspected CVM underwent digital subtraction arteriography, and pre-eclampsia and eclampsia were diagnosed according to the American College of Obstetricians and Gynecologists criteria^18^. Patients with bleeding caused by brain trauma were excluded from the sample. The clinical characteristics of the patients, including maternal age, parity, gestational age, aetiology, Glasgow score at admission, mode of delivery, treatment method, and obstetrical perinatal outcome, are shown in Table 1.

Based on the clinical data of these patients and according to their cerebral bleeding treatment method, they were divided into surgery and conservative treatment groups. Doctors used surgical methods to treat cerebral bleeding disorders in 20 cases, including haematoma evacuation, ventricular puncture and drainage, and others. The remaining patients received conservative treatments, including sedation, reducing the intracranial pressure, and other symptomatic treatments. The surgery and conservative treatment groups were compared with regard to general items to determine whether differences existed. Furthermore, differences between the two groups were compared with regard to maternal mortality and neonatal outcomes, for example, prematurity rate, abortion (including mid-abortion) rate, full-term delivery rate, stillbirths, birth weight, neonatal mortality, and the average Apgar score of newborns. Global outcome at discharge was assessed using the modified Rankin Scale (mRS) 10, with 0–2 defined as a good outcome, 3–6 as a poor outcome, and 6 as death.

Patients with cerebral haemorrhage were divided into the pre-eclampsia (including HELLP syndrome) and other aetiology groups based on aetiology. The data and prognoses are shown in Table 3.

Statistical procedures were performed using SPSS version 20. Differences between two groups with a normal distribution were analysed by unpaired Student's t- tests. Yates' corrected chi-squared test was used for comparisons between categorical data. Univariate logistic regression analysis was performed to examine predictors of poor outcome and maternal death. The predictors were chosen because they frequently appear in case reports of pregnancy-associated ICH: gestational age, Glasgow score, history of neurosurgery, pre-eclampsia (including HELLP syndrome), and O-D time. *P* < 0.05 was considered statistically significant.

## Author Contributions

Z.W.L. designed the study, carried out the statistical analyses and wrote the manuscript. W.L.G., L.M.F. and L.L. participated in the data collection and analysis. Z.W.L. and L.L. revised the manuscript. All the authors read and approved the final manuscript.

## Figures and Tables

**Table 1:** Clinical characteristics and pregnancy outcomes of pregnancy-associated intracranial haemorrhage patients in this sample (N = 39)

Variable	Value	Variable	Value
Age (years)	27.13 ± 5.73	Obstetric outcome: Mortality	30.77% (12)
Gestational age (weeks)	29.42 ± 8.82	Perinatal outcome	
Gravidity	1.87 ± 1.26	Abortion & induced labour	30.77% (12)
Parity	0.31 ± 0.57	Prematurity	41.03% (16)
Glasgow score	11.77 ± 4.51	Term delivery	20.51% (8)
O-D time (hours)	129.79 ± 270.12	Stillbirth	7.69% (3)
mRS	2.16 ± 2.48	Mortality	10.26% (4)
Aetiology		Neonate weight (*g*)	2628.15 ± 889.56
AVM	41.03% (16)	Average Apgar score of newborns	8.11 ± 2.42
AN	10.26% (4)		
MMD	10.26% (4)	Mode of delivery	
Pre-eclampsia	20.51% (8)	Caesarean section	94.87% (37)
Cavernous angioma	5.13% (2)	Vaginal delivery	5.13% (2)
CVST	5.13% (2)	Initial symptoms	
Tumour	5.13% (2)	Headaches	58.97% (23)
Unknown	2.56% (1)	Nausea/vomiting	15.38% (6)
Gestational age		Convulsion	25.64% (10)
Early pregnancy	7.69% (3)	Disturbed consciousness	66.67% (26)
Second trimester	28.21% (11)	Visual disturbance	10.26% (4)
Third trimester	58.97% (23)	Paralysis	25.64% (10)
Puerperium	5.13% (2)	High fever	2.56% (1)

*Abbreviations*: AN, aneurysm; AVM, arteriovenous malformation; CVST, cerebral venous sinus thrombosis; MMD: moyamoya disease; mRS: modified Rankin Scale; O-D time, onset-to-diagnosis time.

*Notes*: Values are the mean ± standard deviation (s.d.).

**Table 2:** Admission data and prognoses of the surgery and conservative treatment groups

Variable	Surgery group (*n* = 20)	Conservative treatment group (*n* = 19)	*P*
Age (years)	26.95 ± 5.20	26.61 ± 5.75	0.27
Gestational age (weeks)	29.89 ± 7.18	30.00 ± 9.82	0.26
Gravidity	2.00 ± 1.41	1.72 ± 1.13	0.68
Parity	0.30 ± 0.57	0.33 ± 0.59	0.83
Glasgow score	11.15 ± 4.88	12.28 ± 4.18	0.53
Obstetric outcome: Mortality	25.00% (5)	17.95% (7)	0.15
Perinatal outcome:			
Abortion & induced labour	30.00% (6)	31.58% (6)	0.14
Prematurity	40.00% (8)	42.11% (8)	0.44
Term delivery	20.00% (4)	21.05% (4)	0.52
Intrauterine death	10.00% (2)	5.26% (1)	0.54
Mortality	5.00% (1)	15.79% (3)	0.27
Neonate weight (*g*)	2498.57 ± 829.05	2767.69 ± 936.96	0.31
Average Apgar score of newborns	7.69 ± 2.45	8.50 ± 2.10	0.35

*Notes*: Values are the mean ± s.d.

**Table 3:** Admission data and prognoses of the pre-eclampsia and other aetiology groups

Variable	Pre-eclampsia group (*n* = 8)	Other aetiology group (*n* = 31)	*P*
Age (years)	28.38 ± 7.82	26.81 ± 5.17	0.75
Gestational age (weeks)	35.25 ± 4.10	27.75 ± 9.14	0.03
Gravidity	2.88 ± 1.96	1.61 ± 0.88	0.12
Parity	0.38 ± 0.74	0.29 ± 0.53	0.96
Glasgow score	8.00 ± 6.00	2.74 ± 3.55	0.02
Obstetric outcome: Mortality	8.75% (7)	16.13% (5)	0.00
Perinatal outcome:			
Abortion & induced labour	0% (0)	38.71% (12)	0.00
Prematurity	37.50% (3)	41.94% (13)	0.56
Term delivery	50.00% (4)	12.90% (4)	0.52
Stillbirth	12.50% (1)	6.45% (2)	0.37
Mortality	50.00% (4)	0% (0)	0.01
Neonate weight (*g*)	2496.88 ± 926.14	2683.42 ± 839.62	0.59
Average Apgar score of newborns	6.28 ± 2.77	8.63 ± 2.10	0.04

*Notes*: Values are the mean ± s.d.

**Table 4:** Univariate logistic regression analysis demonstrating the relationship between poor outcome and other variables

	Poor outcome		Maternal mortality	
	Odds ratio (95% CI)	*P*	Odds ratio (95% CI)	*P*
Gestational age	0.85 (0.69–1.04)	0.10	0.99 (0.91–1.08)	0.81
Glasgow score	1.45 (0.99–2.12)	0.06	1.20 (0.84–1.72)	0.31
History of neurosurgery	73.88 (0.73–7530.79)	0.07	2.17 (0.28–17.19)	0.46
Pre-eclampsia (including HELLP syndrome)	716.33 (1.15–448233.30)	0.04	1.48 (0.08–26.91)	0.80
O-D time	3.55 (1.32–9.55)	0.01	1.73 (1.07–2.91)	0.03

*Abbreviations*: O-D time, onset-to-diagnosis time (*hours*); poor outcome: modified Rankin Scale (mRS) ≥ 3.
